# Lessons learned during a 12-year monitoring project with the endangered Magdalena River turtle (*Podocnemis lewyana*): hunting pressure, habitat degradation, and methodological considerations

**DOI:** 10.1007/s10661-024-12944-0

**Published:** 2024-08-30

**Authors:** Vivian P. Páez, Brian C. Bock, Felipe A. Toro-Cardona, Viviana M. Cartagena-Otálvaro

**Affiliations:** 1 Instituto de Biología, Grupo Herpetológico de Antioquia, Universidad de Antioquia, Calle 70 N° 52-21, Medellín, Colombia; 2 Laboratorio de Ecología y Evolución de Vertebrados, Instituto de Biología, Universidad de Antioquia, Calle 70 N° 52-21, Medellín, Colombia

**Keywords:** Body condition index, Capture frequency, Colombia, Habitat degradation, Hunting, Podocnemididae

## Abstract

Turtle species in the Family Podocnemididae, including the Colombian endemic and critically endangered Magdalena River Turtle *Podocnemis lewyana*, characteristically present low recapture rates that preclude estimation of population parameters using maximum likelihood modeling. In our 12-year monitoring project with this species, we evaluated changes in relative abundances, proportions of sex/size classes, and individual body sizes and body conditions in a population in four channels in the middle Magdalena River drainage. We also inspected for associations between trends in changes in these variables and differences in hunting pressure and habitat degradation. To inspect for temporal and spatial demographic dynamics, we estimated variation in relative abundances using the Catch Per Unit Effort index, the total number of turtles captured over an entire 5-day sampling period using ten baited funnel traps. Relative abundances and the proportions of sex/size classes were different between sites and years. We found a significant decline in the proportion of females and juveniles over time, along with evidence that the females still present were smaller in body size. Our results support the hypothesis that hunting eliminates adult females from these sites, perhaps also translating into a reduction in recruitment. The lack of evidence of generalized declines in body condition of all size classes suggests that habitat degradation might contribute less to the population declines in this region. Our results also illustrate that even when recapture rates are low, monitoring turtles via standardized trapping may yield insights into the population’s conservation status that other relative abundance indices cannot.

## Introduction

Comprehensive estimations of population size trends across all sex/size classes in non-sessile animals can be time-consuming and expensive (Greenwood & Robinson, 2006). In addition, the accuracy of these estimates depends in part on the ability to correct for spatiotemporal and class differences in detection probabilities (imperfect detection) and on the consistency with which the methodology is employed (Pollock et al., 2002). However, when an effective and methodologically constant and unbiased technique is used, such as Capture-Mark-Recapture (CMR), this permits the estimation of several fundamental population parameters in addition to population size, such as annual survival rates for each class as well as transition probabilities between them (Bailey et al., 2004). With CMR, detection probabilities for each sex/size class can be interpreted as the probability of capturing an individual within the population when it is present. Unfortunately, obtaining adequate CMR data is difficult for rare species with low and heterogeneous detection probabilities, such as some aquatic turtles (Banks-Leite et al., 2014; Dreslik et al., 2005). This is generally true for turtle species in the Family Podocnemididae due to a combination of biological characteristics of these species, such as their low capture rates and high vagilities (Fachín-Terán & Vogt, 2004; Fachín-Terán et al., 2003; Bernhard & Vogt, 2012; Bernardes et al., 2014; Portelinha et al., 2014; Páez et al., 2015; Leão et al., 2019), complex use of different habitat types by individuals of each sex/size class, which may vary by hydrological season (Fachín-Terán et al., 2005; Naveda-Rodríguez et al., 2018; Alzate-Estrada et al., 2020), their primarily herbivorous diets that reduce the efficiency of trap baiting (Eisemberg et al., 2017; Fachín-Terán et al., 1995; Ferrara et al., 2017), and their rarity due to human impacts that have driven populations to critically low numbers (Arraes et al., 2016; Forero-Medina et al., 2021; Páez et al., 2015). Because of these factors, few publications have been able to estimate the detection probabilities, absolute densities, annual survival rates, and transition probabilities using CMR demographic data from their studied Podocnemidid population. Two such studies have been conducted on the endangered *Podocnemis expansa* in the Orinoco drainage of Venezuela (Mogollones et al., 2010; Peñaloza Chacín, 2010).

The other Podocnemidid species for which a study was able to successfully estimate these parameters was conducted on the critically endangered *Podocnemis lewyana* in the middle Magdalena River drainage of Colombia (Páez et al., 2015). In that study, a Pradel model (Pradel, 1996) was used with a maximum likelihood modeling approach with the software MARK (White & Burnham, 1999) to estimate annual population change, transition probabilities, and detection probabilities by sex/size classes. The study also conducted a retrospective analysis of the realized population growth rate during the study and made a population matrix projection of likely future trends based on these estimates. Both analyses indicated that this population was declining at a rate of approximately 9% annually and that the overall detection probability was, on average, 0.059 (range = 0.010–0.145); that is, only around 6% of the individuals within the population were captured.

In the present study, we have continued monitoring this declining *P. lewyana* population for an additional 8 years. However, our more limited funding affected our ability to employ the same frequency and spatial sampling intensity, resulting in lower recapture rates that precluded our using the MARK software for our analyses (because estimates were unobtainable or unreliable). Like many other CMR studies that suffer from low recapture rates, we had to resort to the use of a relative abundance index for our analyses in this study. All such indices comprise some count of the individuals or their signals as the numerator and some measure of the sampling intensity as the denominator to document or compare the spatiotemporal tendencies of the relative abundance of one or more populations, assuming the index correlates with the actual population size. Even if we do not know the exact proportional relationship between the index and the true abundance of the population, if the sampling method and effort are consistent, these indices are helpful in tracking the changes in the population abundance (Sinclair et al., 2006).

In Podocnemidid turtles, some relative abundance indices employed have been annual counts of clutches on nesting beaches (Andrade et al., 2022), counts of nesting females (Forero-Medina et al., 2021), shoreline censuses of basking and floating individuals (Conway-Gómez, 2007; Yapu-Alcázer et al., 2018), or measures of trapping success rates, usually expressed as turtles per trap-hour (Fachín-Terán & Vogt, 2004; Fachín-Terán et al., 2006; Bernardes et al., 2014; Portelinha et al., 2014; Páez et al., 2015; Alzate-Estrada et al., 2020; Sepúlveda-Seguro et al., 2020). For this study, the low recapture rates also led us to adopt a relative abundance index approach to monitoring these sites during all 12 years of fieldwork. While doing so, we also attempted to assess the impact of two potential causes of the previously identified decline: continued illegal harvest of turtles in this area and continuing habitat degradation (deforestation) along the shorelines of four channels of the Magdalena and Carare rivers, Colombia. Hunting pressure is selectively directed towards larger individuals, especially adult females, who are particularly vulnerable during the nesting season when they leave the channels to nest on river beaches (Páez & Bock, 2012; Páez et al., 2012, 2015). We therefore inspected for changes in the proportion of individuals in the different sex/size classes over the 12 years. The other potential factor, habitat degradation due mainly to the expansion of cattle ranching and planting of agricultural crops along shorelines, might increase water contamination and reduce food availability and basking opportunities and thus might be expected to affect equally individuals of all size classes, or at least of both sexes within each size category. We, therefore, also quantified the extent of natural vegetation loss in our study area during these 12 years. Finally, to inspect whether this habitat degradation was adversely affecting the turtles, we calculated a Body Condition Index (BCI) for each turtle captured each year. The BCI was intended to quantify the relative body weights of the turtles while controlling for differences in body sizes, with a decrease in the BCI values suggesting the individuals are environmentally stressed and, therefore, lighter. Here, we report on variation within and among the four study channels in the middle Magdalena River drainage from 2012 to 2023 in terms of relative abundances, population structure (annual proportion of each sex/size class in the population), and turtle body sizes and conditions. We also make recommendations on how best to study aquatic turtle populations using relative abundance indices when the estimation of population size or other demographic parameters using rigorous CMR methods is not feasible due to low re-capture rates.

## Materials and methods

### Sites and habitat characteristics

**Fig. 1:**
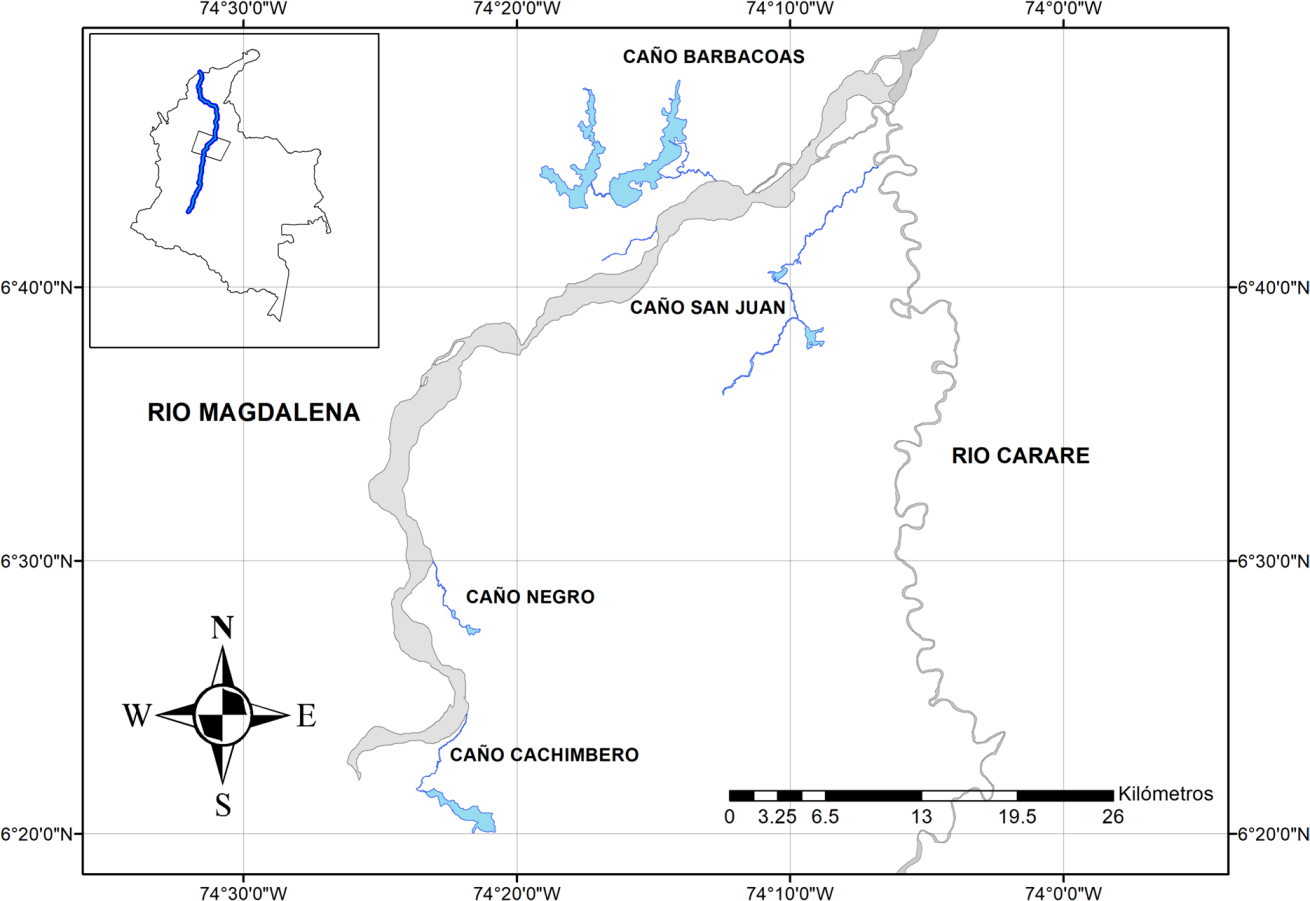
Map of the study area showing the location of the four channels (Caño Cachimbero, Caño Negro, Caño San Juan, and Caño Barbacoas) where *Podocnemis lewyana* was monitored from 2012 to 2023

The data on *Podocnemis lewyana* in this study were collected in four channels that connect wetland areas to the Magdalena and Carare rivers in the municipalities of Yondó (Antioquia department) and Cimitarrá (Santander department), Colombia (Fig. 1). Caño Negro (CN) is a channel located in Cimitarrá and connects the La Chiquita and El Encanto wetlands to the Magdalena River. Caño Cachimbero (CC) is a channel in Cimitarrá that connects the Cachimbero wetland to the Magdalena River. Caño Barbacoas (CB) is a channel in Yondó that connects the Barbacoas and Ciénega Grande wetlands to the Magdalena River. Finally, Caño San Juan (CSJ) is a channel located in Cimitarrá and connects the La Duda wetland to the Carare River just before its confluence with the Magdalena River. Páez et al. (2015) and Alzate-Estrada et al. (2020) describe in more detail these four channels in terms of their physical characteristics, vegetative covers, proximity to suitable nesting habitat, and levels of anthropogenic intervention. The size (length, width, and depth) of the channels increases in the order CN < CC < CB < CSJ.

This region has a bimodal precipitation regime that exhibits two dry seasons, or low-water seasons (Bock et al., 2021; Season 1 = December to March; Season 3 = July to September) and two rainy seasons, or high-water seasons (Season 2 = April to June; Season 4 = October to November). The reproductive cycle and movement patterns of *P. lewyana* individuals are influenced by these hydrological fluctuations and vary depending on the sex and size class of the individuals (Alzate-Estrada et al., 2020; Páez et al., 2015).

### Sampling design

From 2012 to 2014, we conducted an intensive monitoring study that was standardized by effort in the four channels and consisted of visiting each channel at least once in each of the four hydrological periods (Seasons 1 to 4) (Páez et al., 2015). Each sampling event consisted of 5 consecutive days of trapping, placing 10 double funnel traps (80 cm external diameter and 2.6 m length, with a mesh size of 5 cm^2^) approximately 400 m apart in the channel being sampled. Traps were baited with leaves of the local plant *Maclura tinctoria*, left open 24 h/d, and checked every 4 h from 0500 to 2300 h. Here, we refer to this period of the monitoring project as Period I (intensive sampling).

Budget limitations caused us to reduce the intensity of sampling in subsequent years. From 2015 to 2016, we were able to conduct sampling events at only three of the four sites (CSJ, CB, and CN) and in only one or two of the four hydrological periods. In 2018, we could resume sampling events in all four hydrological periods but only in two of the four channels (CN and CSJ). In our monitoring project, we refer to this less intense monitoring period as Period M (moderate sampling). Finally, in 2020, 2022, and 2023, we could sample only one site (CSJ) in only one of the four hydrological seasons. We refer to this final period of our monitoring project, Period L (low sampling). During the entire 12-year monitoring, some traps were damaged or stolen, or the sampling had to be prematurely terminated due to climatological conditions (sudden changes in the river levels that precluded our ability to set the traps) or sociopolitical considerations (such as aggressive interactions between the illegal armed groups or narco-guerrilla and the military forces or threats to us from the local/immigrant common thieves). These sampling periods were excluded from the analyses to ensure sampling intensity was held constant and thus would not affect the probability of detection of turtles.

### Relative abundance indices

Over all three periods from 2012 to 2023, we conducted 79 sampling events; that is, 395 trapping days or 94,800 trap h (395 days × 24 h × 10 traps). In this study, we considered two indices of relative abundance. First, the Catch Per Unit Effort (CPUE) index, traditionally used with freshwater turtle species, is expressed in terms of captures per trap-day (10 trap-days of effort each sampling day) to facilitate comparison with other studies. Second, we also calculated a combined CPUE index, which included all turtles captured over a 5-day sampling period (50 trap-days). We use this second CPUE index to eliminate the bias in the first one (captures per trap-day) due to a lack of independence between sampling days within a 5-day sampling period (see Results). Our two CPUE indices allowed us to inspect for variation in relative abundances among channels and between seasons and years.

### Morphometrics

The captured turtles were given individual marks by notching marginal scutes (Cagle, 1939). All individuals were measured, weighed, photographed, and released at their capture sites. Straight-line carapace length (SCL) for small individuals was measured with a Mitutoyo digital caliper with a precision of ± 0.01 mm, and larger individuals were measured with a mechanical caliper with a precision of ± 0.1 mm. Weight also was quantified, depending on the size of the turtle, with one of two Pesola scales. Smaller individuals were weighed with a 1 kg scale with a precision of ± 0.1 g and larger individuals were weighed with a 20 kg scale with a precision of ± 200 g. We classified all individuals into sex/size classes (Páez et al., 2015). Adult and sub-adult individuals (but not juveniles) were classified for sex based on the shape of the anal scute and dimorphism in head coloration (Páez et al., 2009, 2012). Males were classified into two size classes: small males (SM), which were males with a SCL > 15 cm and < 24.9, and large males (LM) with a SCL ≥ 25 cm. Our SM class contained both sub-adult and mature males (minimum size at reproduction for males has been estimated at approximately 20 cm SCL; Páez et al., 2015). Females were also classified into two size classes: small females (SF), which were females with a SCL > 15 cm and < 29.9 cm, and large females (LF) with a SCL > 30 cm. The SF class consisted entirely of sexually sub-adult females (minimum size at reproduction for females has been estimated at approximately 30 CM SCL; Páez et al., 2015). Individuals too small (SCL < 15 cm) with a lack of clear dimorphic characteristics were classified as juveniles.

We calculated the Body Condition Index (BCI) for every turtle using the residuals of the regression of log-transformed body mass vs log-transformed SCL to inspect the linearity of the relationship and homogeneity of variances about the regression line. Green (2001) discussed the biological and statistical assumptions underlying the use of such residuals as an index of body condition and stressed the need to examine first whether the body mass/body size relationship is truly linear. Green (2001) also suggested the use of statistical alternatives to ordinary least squares regression to avoid violating certain statistical assumptions in this method, but Schulte-Hostedde et al. (2005) compared these alternative methods with the traditional least squares regression method and found they did not perform significantly better. Therefore, we used ordinary least squares regression to generate residual values for every turtle as our BCI. We also report details on the linearity of the relationship obtained with our data.

### Hunting pressure at the sites

In 2014 (the last year when all four channels were sampled), we attempted to quantify the levels of hunting pressure in all four channels using two methods. The first was to record the number of times we saw local fishermen in each channel while conducting fieldwork. Fishermen who capture *P. lewyana* individuals in their nets while fishing (by-catch) almost always harvest them (VPP and VCO, pers. obs.). Because we sampled along differing lengths of the different-sized channels, we converted the total number of fishermen seen in each channel to a per km index. The second method to quantify levels of hunting pressure at a site was to conduct consented interviews with five local fishermen at each site. Questions assess whether they consumed turtles (meat and/or eggs), commercialized turtles (adults and/or eggs), or perceived the local turtle population as stable or declining, and in the latter case, to what they attributed the decline.

### Quantification of rates of habitat loss

To estimate land cover changes during the sample time (2012–2023), we used Landsat 7 and Landsat 8 satellite images for each year taken during the dry season (January to March). We used QGIS to fix the Landsat 7 scanline error and then used the RStoolbox R package (Leutner et al., 2017) to make the atmospheric correction for all images using the Dark Object Subtraction method (Chavez, 1988) and to calculate the Tasseled Cap Transformation (Kauth & Thomas, 1976) to improve the classification process (Dymond et al., 2002; Healey et al., 2005; Insaurralde, 2019). We performed a supervised classification and land change analysis only for the CN and CSJ channels because the total time period sampled was the same for both sites. First, we used a 2 km buffer around both channels to restrict the analysis area according to the terrestrial dispersal capacity of the species. Then for each channel, we created training polygons for three classes each year: water bodies (rivers, channels, and ponds); transformed vegetation (crops and pastures); and conserved natural vegetation (forest). We classified the images using the random forest algorithm with the randomForest R package (Weiner, 2002) by randomly selecting 80% of the training polygons and then evaluating the classification accuracy with the Kappa index, using the other 20% of the data. To quantify changes in land cover between sampling years, we used the OpenLand R package (Exavier & Zeilhofer, 2021), focusing on the transitions from conserved to transformed landscape. Also, we calculated the accumulative loss of conserved vegetation each year for both sites by estimating the new lost area in a transition time.

### Statistical analyses

To inspect for lack of independence among sampling days within each 5-day sampling event, we calculated the total number of turtles captured on the first to the fifth day of trapping for all 79 sampling events and conducted a regression analysis on these totals (total number of turtles trapped for each day during the sampling events). We also calculated regression analyses separately for each of the 79 sampling events. The power of these latter tests was low due to the small sample size (5 data points), but we could inspect the tendencies (slopes) in each resulting regression equation to examine how many exhibited increasing numbers of turtles captured each successive day (positive slopes) vs. declining numbers of turtles captured each successive day (negative slopes).

We regressed log-transformed body weight on log-transformed SCL to inspect the linearity of the relationship and homogeneity of variances about the regression line. We used Anova tests on the residuals from this regression to inspect for differences in BCI among sex/size classes and channels. Anovas were also used to inspect for differences among channels in mean SCL and body mass of individuals.

We inspected for changes in relative abundances in the four channels over the course of the study by regressing the “day of the study” when a 5-day sampling period began vs. the relative abundance estimate obtained. We inspected for changes in SCL, body weight, and BCI during the study using Anova analyses (data pooled from all sampling seasons in each channel each year). To inspect for changes in the proportion of individuals in the different sex/size classes over time, we used χ^2^ tests comparing frequencies of individuals in each class for each Period (I, M, and L) for channels with data from two or more of these periods.

## Results

During the 79 sampling events, with the simultaneous use of 10 baited double funnel traps, we captured a total of 934 *Podocnemis lewyana* individuals. On first capture, 395 of them were juveniles (42.3%), 128 were SM (13.7%), 64 were LM (6.9%), 224 were SF (23.9%), and 123 were LF (13.2%). In this study, we did not consider recapture events that occurred when the same individual was captured twice within any 5-day sampling period. Of the 934 individuals we captured in our study, 879 were only captured once, 55 were recaptured one time, and 6 of these were recaptured a second time, for a total of 995 capture events and an overall recapture rate of 5.9%. Of the 55 individuals recaptured, 22 had transitioned to a larger size class when recaptured. The rate of capture and recapture of these individuals varied over the three sampling periods. During the 3-year intensive sampling period (2012–2014), 631 individuals were captured (67.5% of the total for the study), with a recapture rate of 7.1% of these individuals. In contrast, during the moderate sampling period (2015–2018), only 207 new individuals were captured (22.2% of the total for the study), with a recapture rate of only 3.4%. Similarly, in the low sampling period (2020–2023), only 96 new individuals were captured (10.3% of the total), with a recapture rate of 3.1%.

Over the entire study, we captured or recaptured 233 turtles on the first day of the sampling period, 217 on the second day, 189 on the third day, 204 on the fourth day, and 152 on the last day (*r* =  − 0.90, *P* < 0.05). Of the 79 correlation coefficients calculated for each of the separate 5-day sampling periods, 52 (64.6%) had negative slopes, five at *P* < 0.10 and two at *P* < 0.05 (vs. only three at *P* < 0.10 for positive slopes). This trend of declining detectability over sampling days indicated a lack of independence between days, which is why we chose to only use as our CPUE index the total number of turtles captured during a 5-day sampling session for the remaining analyses. The average relative abundance over all the years and channels was 12.6 CPUE ± 7.5 CPUE (range, 2–44 CPUE); that is, on average, we captured 2.52 turtles/day with the use of 10 traps or 0.0105 turtles/trap-h.

**Fig. 2:**
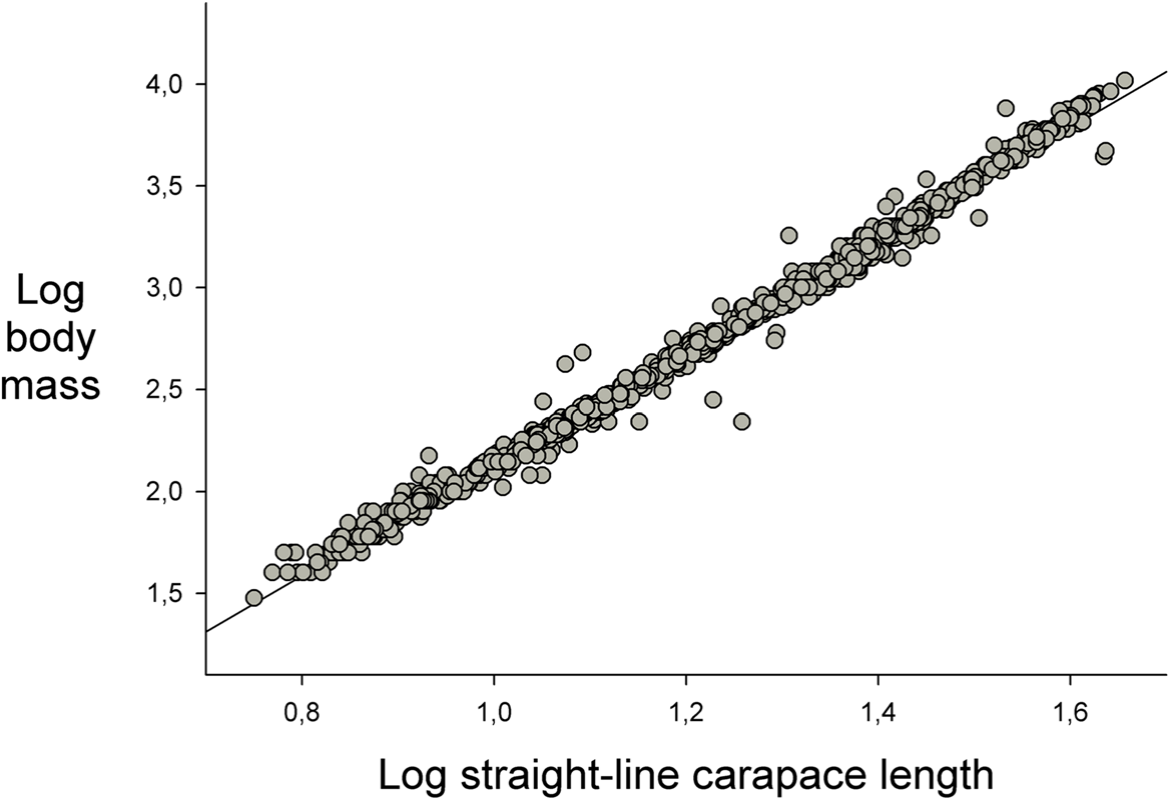
Regression of Log straight-line carapace length on Log body mass (Log body mass =  − 0.615 + 2.751 × Log straight-line carapace length) of the *Podocnemis lewyana* individuals captured during the monitoring project. The residuals of this regression were used as a Body Condition Index

Log transformed body size (SCL) and weight variables were linearly related (*R*^2^ = 0.9925, *F*_(1, 993)_ = 132057, *P* < 0.001) with homogeneous variances around the regression line (Fig. 2), so we used the residuals from this regression line as our BCI. Over the 12-year monitoring period (data from all channels pooled), we compared the BCI values of individuals belonging to the five different sex/size classes and found a significant difference (Anova, *F*_(4, 990)_ = 27.89, *P* < 0.001), because large females exhibited BCI values significantly greater than individuals in the other four sex/size classes.

There were marked differences among the four channels regarding their population structures (*χ*^2^ = 394.2, df = 12, *P* < 0.001). Smaller individuals (juveniles, SM, and SF) predominated in the two narrower, shallower channels. In CC, we captured no large females and only one large male, while in CN only one large female was captured. Considering only juveniles and small adults of both sexes, CN contained significantly more small adults than CC (*χ*^2^ = 14.8, df = 2, *P* < 0.001). In contrast, in the two wider, deeper channels, all sex/size classes of turtles were captured in substantial numbers. These two wider channels contained similar proportions of juveniles, but CB contained more males (individuals in the SM and LM classes), while CSJ contained more females (individuals in the SF and LF classes) (*χ*^2^ = 56.4, df = 4, *P* < 0.001). Finally, the differences in population structure among the channels translated to significant differences among channels in mean SCL and mean body weight (SCL, *F*_(3, 991)_ = 151.1, *P* < 0.001; weight, *F*_(3, 991)_ = 73.5, *P* < 0.001). Relative abundances also differed among the channels, in the following order: CB (mean CPUE = 8.6), CC (mean CPUE = 9.6), CN (mean CPUE = 11.2), and CSJ (mean CPUE = 17.2).

**Fig. 3:**
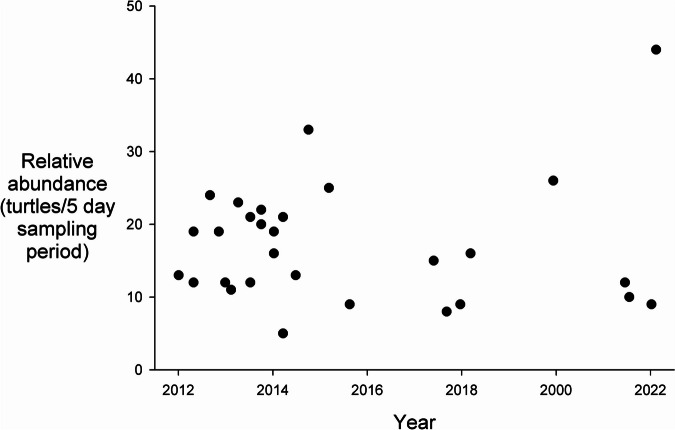
Temporal variation in the Relative Abundance index (number of turtles captured per 5-day sampling period) at the Caño San Juan site (the channel with the largest sample size) over the course of the monitoring study. There also were no significant trends in relative abundance in the other three channels

**Fig. 4:**
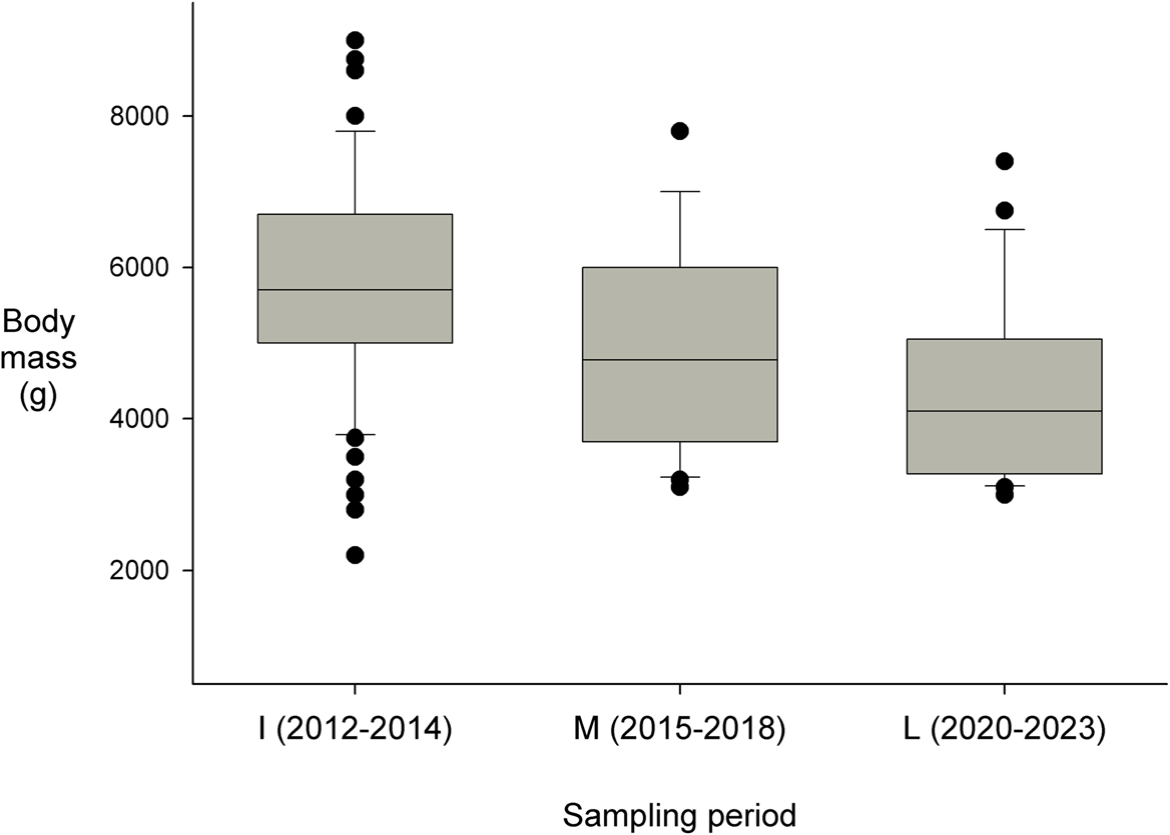
Changes in mean body size of large female individuals of *Podocnemis lewyana* in the Caño San Juan channel in the three periods during the monitoring study

There were no trends apparent in the relative abundance estimates over time in any of the four channels (regressions, all *P*’s > 0.10; Fig. 3, Table 1). However, population structure (the proportion of individuals within the five sex/size classes in the channel) varied over the three periods of the monitoring study in three of the four channels (CB, *χ*^2^ = 11.27, *P* < 0.05; CN, *χ*^2^ = 8.35, *P* < 0.05, CSJ, *χ*^2^ = 50.20, *P* < 0.001; the CC site was only monitored during Period I). The differences over time in the channels were due to a decrease in the proportions of the populations comprised of large adults (LM and LF), and a decrease in the proportion of juveniles (Table 2). Disregarding juvenile individuals (too small to sex), the body sizes of the turtles in the channels did not vary among the sampling periods, except in the narrow CN, where LF increased in body size from Period I to M (SCL, F_1,36_ = 4.92, *P* < 0.05; mass, F_1,36_ = 6.97, *P* < 0.05) and in the wide channel CSJ, where LF decreased in body size from Period I to Period M and L (SCL, F_2,276_ = 10.18., *P* < 0.001; mass, F_2,276_ = 14.46, *P* < 0.001; Fig. 4). There also was a (non-significant) trend for Juveniles in this channel to be larger in the latter sampling periods.

**Table 1:** Relative abundances (mean number of individuals captured during 5-day sampling periods) of *Podocnemis lewyana* individuals in the four channels of the Magdalena and Carare rivers, Colombia, monitored from 2012 to 2023 (*I* intense sampling, *M* moderate sampling, *L* low sampling)

Channel	I_2012–2014_	M_2015–2018_	L_2020–2023_	Mean
Caño Barbacoas	9.2	6.3		8.6
Caño Cachimbero	9.6			9.6
Caño Negro	10.2	13.7		11.2
Caño San Juan	16.6	16.4	20.2	17.2

**Table 2:** Changes in the proportions of individuals in each sex/size class during the monitoring of the four channels of the Magdalena and Carare rivers, Colombia, from 2012 to 2023 (*J* juveniles, *SM* small males, *LM* large males, *SF* small females, *LF* large females)

Channel	Sex/size class				
Caño Barbacoas	J	SM	LM	SF	LF
I_2012–2014_	0.25	0.32	0.15	0.16	0.12
M_2015–2018_	0.00	0.68	0.11	0.11	0.11
Caño Cachimbero	J	SM	LM	SF	LF
I_2012–2014_	0.88	0.02	0.01	0.10	0.00
Caño Negro	J	SM	LM	SF	LF
I_2012–2014_	0.70	0.12	0.04	0.13	0.01
M_2015–2018_	0.73	0.04	0.01	0.22	0.00
Caño San Juan	J	SM	LM	SF	LF
I_2012–2014_	0.29	0.12	0.10	0.24	0.24
M_2015–2018_	0.21	0.13	0.06	0.41	0.19
L_2020–2023_	0.10	0.14	0.01	0.58	0.17

### Hunting pressure

In 2014, we encountered the following mean daily “densities” of fishermen present in the four channels: CB = 6.46 fishermen/km, CN = 1.00 fishermen/km, CC = 1.08 fishermen/channel, and CSJ = 0.48 fishermen/km. In all four sites, respondents to the interview confirmed there was a strong cultural tradition of harvesting *P. lewyana*. Over 70% of respondents admitted to both consuming and commercializing *P. lewyana* (meat and eggs). All agreed that *P. lewyana* abundance had declined in their sites; 70% expressed the opinion that this was due to overharvesting, while the remaining 30% responded it was due to the combination of overharvesting and habitat degradation (deforestation and water contamination).

### Habitat changes

**Fig. 5:**
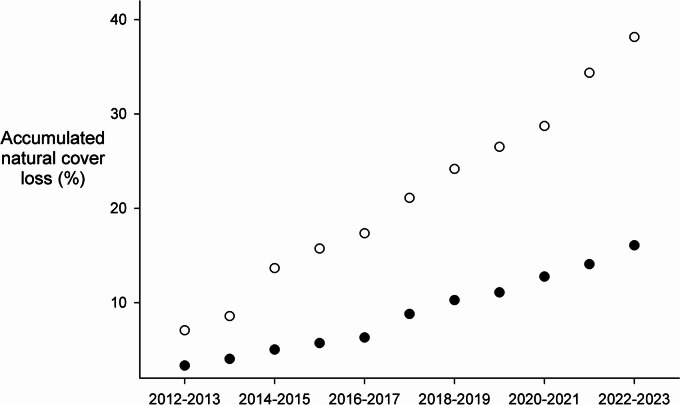
Accumulated loss of the natural vegetative cover originally present along the shorelines of the Caño Negro and Caño San Juan channels over the course of the monitoring study. Solid circles = Caño Negro, Open circles = Caño San Juan

The image classification analysis exhibited good evaluation metrics, with mean kappa values of 0.98 and 0.97 for CN and CSJ, respectively. The land cover transformation analysis conducted over the sampling period showed a similar trend of the average annual increase in transformed vegetation. CSJ showed a mean annual increase of 9.1% in transformed vegetation, while CN only exhibited an 8.7% increase (Fig. 5). However, at the start of our study (2012), the amount of transformed land already present in CN was substantially more extensive than in CSJ. Our results estimated a total forest loss of approximately 1605 ha for CN and 3814 ha for CSJ during the study (Fig. 4). However, CN showed a trend of maintaining a similar transformation rate in the last 2 years of the study, while CSJ shows a trend of increasing rates of forest loss.

## Discussion

The low overall recapture rate in this study (5.9%) is typical of CMR studies with Podocnemidid turtles, which range from low recapture probabilities of 3.1–6.0% (Fachín-Terán & Vogt, 2004; Fachin-Terán et al., 2006; Bernardes et al., 2014; Portelinha et al., 2014; this study), to slightly higher recapture probabilities of 7.4–11.8% (Bernhard & Vogt, 2012; Páez et al., 2015; Sepúlveda-Seguro et al., 2020). This represents a problem for researchers attempting to document demographic declines or demonstrate recoveries resulting from management efforts. In the early years of this monitoring project, we had the funds and personnel necessary to intensely sample each of the four channels one or more times in each of the four hydrological seasons each year, with sufficient recapture rates (8.7%) to permit the use of a Pradel model and maximum-likelihood modeling approach to estimate population parameters and tendencies (Páez et al., 2015). The evidence from these analyses that demonstrated population declines in our study channels led us to continue our monitoring efforts there, but the reduced intensity of sampling in later years precluded additional modeling efforts due to the lower recapture rates. In software packages such as MARK (White & Burnham, 1999), when recapture rates are too low, analyses tend to not support models showing differences in survival rates or detection probabilities among sites, sexes, or size classes, limiting their utility. For this reason, we adopted an approach for analyzing our overall 12-year data set that was based on relative abundance indices.

Most studies of Podocnemidids that report relative abundance indices quantify it with a CPUE index expressed in terms of turtles captured per hour or day or turtles captured per trap-hour or trap-day. As expected, these daily capture indices vary considerably between *Podocnemis* species from extremely low values of less than 0.008 captures/trap-h (Sepúlveda-Seguro et al., 2020), intermediate values of from 0.01–0.12 captures/trap-h (Bernardes et al., 2014; Páez et al., 2015; this study) to values of 0.52–0.59 captures/trap-h (Bernhard & Vogt, 2012; Portelinha et al., 2014). Additionally, they vary between capture methods, locations, seasons, and years (Fachín-Terán & Vogt, 2004; Fachín-Terán et al., 2006; Bernardes et al., 2014; Portelinha et al., 2014; Alzate-Estrada et al., 2020; Sepúlpeda-Seguro et al., 2020). However, most studies leave their traps open in the same sites over a number of successive days during each sampling period. An implicit assumption in such studies is that the detection probabilities (capture rates) on each day of a sampling period are independent. Our study produced evidence that this is not the case for *P. lewyana*. Our data showed a tendency for capture rates to decline over time during the 5-day sampling periods, indicating that detection probabilities were not independent over days. The lower capture rates in the later days of a sampling period may have resulted from turtles leaving the area near the traps after being captured, marked, and released (leaving a smaller pool of individuals to sample over subsequent days). It also might have been due to turtles that were not captured also leaving the sampling site due to the disturbances related to our checking the traps repeatedly over the 5-day period. We recommend that future turtle trapping studies inspect for evidence of independence of capture rates over sampling periods, as this is an important assumption for many statistical analyses of capture data. If there is evidence of a lack of independence, we recommend that studies use a CPUE index that pools data collected overall sampling days, as we did in this study.

Páez et al. (2015) showed that during the first 3 years of this study, on average our study sites experienced a 9% annual decline in densities, with our population matrix projections corroborating that this tendency would likely continue. However, our re-analysis of these data combined with additional years of (less intensive) monitoring failed to detect a statistically significant decline using a relative abundance approach. Relative abundance estimates differed significantly among sites but also varied considerably within sites over successive sampling events, probably due to a variety of factors (different microclimatic conditions and/or hydrological conditions during each sampling event, etc.). It would be interesting in future studies to explore more the effects of different factors on relative abundance estimates in this species so that trapping could be conducted in a way to control better for these sources of variation.

Other demographic studies also have shown the difficulties in demonstrating density trends in species where detection probabilities vary greatly among sampling events, or where densities actually fluctuate dramatically over time as part of the natural biology of the species (Hayes & Steidl, 1997).

However, our capture data set did reveal differences in the proportions of individuals in the different sex/size classes, both spatially and temporally. The spatial differences were seen in the proportions of small vs. large individuals in the shallow vs. deeper channels. Substantial numbers of smaller individuals were found in all four channels, so it is probably more accurate to say that larger individuals use shallower channels less, rather than to conclude that smaller individuals “prefer” shallower channels. We also documented temporal changes in the proportion of individuals in the different sex/size classes, but only in the deeper channels where all size classes of individuals occurred in substantial numbers. The evidence that the proportions of large females and juveniles were declining in these sites suggests that hunting pressures targeting larger individuals, especially reproductive females, might have led to a reduction in recruitment in these sites. Our alternative hypothesis for explaining declines in this study was that continuing habitat degradation might be negatively influencing all sexes and/or size classes in the sites. We showed that deforestation in our study sites is an ongoing problem, especially in those sites that contained a greater proportion of natural vegetative cover at the beginning of our monitoring project. However, our attempt to document the effects of habitat change on turtles through the inspection of changes in their body conditions over time was not significant. Large females exhibited higher body condition index values, perhaps because during at least some seasons when they were captured, they were investing in depositing yolk in their eggs, but there were no generalized trends in decreasing body conditions in any sex/size class over time.

Our capture data revealed that the population structure of *P. lewyana* varied depending on the size of the channel sampled. Other studies of freshwater turtle species using trapping methods also have documented spatial differences in population structures (sex ratios, body sizes, proportions of adults vs. juveniles) associated with different habitat features or variations in the severity of different human impacts (Close & Seigel, 1997; Daza & Páez, 2007; Hamer et al., 2016; Marchand & Litvaitis, 2004; Steen & Gibbs, 2004). Our study also revealed temporal changes in the population structure of *P. lewyana* inhabiting the larger channels, with a reduction in the proportion of large females and juveniles over time, presumably due to a preferential harvest of large females and a concomitant reduction in recruitment. Chen and Leu (2009) also showed that a construction project in a river in northern Taiwan led to a reduction in the proportion of large females and juveniles present in the population after the perturbation. Other studies with freshwater turtles also have shown temporal changes in the proportions of different size classes in populations due to the arrival of an introduced predator, or from human perturbations to the habitat, or other unknown causes (Browne & Hecnar, 2007; Nickerson & Pitt, 2012; Usuda et al., 2012; East et al., 2013; Kageyama et al., 2021).

## Conclusions

Although demographic studies of freshwater turtle populations may be challenging, especially for species like those in the Family Podocnemididae where recapture rates are low, trapping studies may still obtain useful data on their populations by using a relative abundance approach. Not only may they show trends in apparent densities over time, but they also yield useful data on the proportions of individuals in the different sex/size classes and on the body sizes and conditions of the turtles captured, data that are not obtained through the use of other sorts of relative abundance indices such as nest counts or shoreline censuses. However, such monitoring studies based on captures should use a standardized methodology throughout the study and test to see whether capture probabilities on successive days of the sampling periods are independent or not. Information obtained on the proportions of individuals in the different sex/size classes and their body conditions also may provide insights into factors affecting the population. Here, our relative abundance index failed to corroborate the statistically significant declines our earlier study suggested are occurring at these sites, but we did show that hunting pressure and habitat degradation are occurring there and that the former probably has a relatively greater negative effect on the persistence of large adult female turtles, precisely the sex/size class with the biggest reproductive value in this population (Páez et al., 2015).

## Data Availability

No datasets were generated or analysed during the current study.
